# A comparative study on nosocomial and community-acquired bacterial urinary tract infections: prevalence, antimicrobial susceptibility pattern, and associated risk factors among symptomatic patients attending Hiwot Fana Comprehensive Specialized University Hospital, Eastern Ethiopia

**DOI:** 10.3389/fepid.2025.1517476

**Published:** 2025-04-07

**Authors:** Sisay Fekadu, Fitsum Weldegebreal, Tadesse Shumie, Getachew Kabew Mekonnen

**Affiliations:** ^1^ School of Medical Laboratory Sciences, College of Health and Medical Sciences, Haramaya University, Harar, Ethiopia; ^2^ Department of Clinical Laboratory, Hiwot Fana Specialized University Hospital, Harar, Ethiopia; ^3^ Laboratory Bacteriology Research, Department of Diagnostic Sciences, Faculty of Medicine and Health Sciences, Ghent University, Ghent, Belgium

**Keywords:** nosocomial acquired UTI, antimicrobial susceptibility patterns, community-acquired UTI, Ethiopia, risk factors

## Abstract

**Background:**

Urinary tract infections (UTIs) remain one of the most common diseases worldwide that occur both in the community and in healthcare settings. Thus, this study aimed to compare the burden of nosocomial and community-acquired bacterial UTIs among patients attending Hiwot Fana Comprehensive Specialized University Hospital, Eastern Ethiopia.

**Method:**

A hospital-based cross-sectional study was conducted using a convenient sampling technique from January 2024 to April 2024. Descriptive statistics were employed, and bivariate and multivariable logistic regression analyses were used to identify associated factors at *p* < 0.05 with a 95% confidence interval (CI) considered statistically significant.

**Results:**

The rate of hospital-acquired UTIs was 42% (95% CI: 35–50), while the rate of community-acquired UTIs was 28% (95% CI: 22–36). The predominant bacterial isolates were *Escherichia coli* (37%), *Staphylococcus aureus* (7.8%), and *Klebsiella pneumoniae* (7.8%). The overall multidrug resistance rate was 91 (77.8%). Lack of formal education [adjusted odds ratio (AOR), 0.02; 95% CI: 0.001–0.6], surgery during admission (AOR, 0.02; 95% CI: 0.002–0.3), delay in voiding urine (AOR, 0.01; 95% CI: 0.005–0.1), previous UTIs (AOR, 0.04; 95% CI: 0.004–0.4), and previous admission (AOR, 0.07; 95% CI: 0.01–0.5) were the main factors significantly associated with bacterial UTIs.

**Conclusions:**

A significantly higher prevalence of hospital-acquired bacterial UTIs was observed compared to community-acquired bacterial UTIs. The commonest isolates were *E.coli*, *S. aureus*, and *K. pneumoniae*. The drug resistance rate was very high. Modifiable individual-level factors were the major significant factors of UTIs. Thus, health workers and other stakeholders should tackle UTIs by increasing community awareness, promoting personal hygiene, and improving healthcare service quality.

## 1 Introduction

Urinary tract infection (UTI) is the most common bacterial infection, and some groups of the population were at increased risk of acquiring the disease, resulting in nearly 7 million hospital visits and 100,000 hospitalizations (1). Urinary tract infections can be classified as complicated and uncomplicated based on the severity of the disease. Complicated urinary tract infection starts from the bladder and extends to the kidneys and is associated with abnormalities, immunosuppression, and previous exposure to antibiotics. Uncomplicated urinary tract infection involves the lower urinary tract, specifically the bladder and urethra (2). UTIs can be categorized based on their site of acquisition: nosocomial urinary tract infection is acquired in a healthcare facility or during patient care, and normal flora residing in the body are related to risk factors (3), whereas community-acquired urinary tract infection (CAUTI) is acquired outside of a healthcare facility or within 48 h of admission (4).

According to a global study conducted, ∼404.4 million people were diagnosed with urinary tract infections, resulting in 236,786 deaths (5). Similar global and regional studies showed *E. coli* and *K. pneumoniae* accounted for 40% and 10% of global antimicrobial resistance-related and attributed deaths in UTIs, respectively (6). A study conducted in Kuwait showed significant bacteriuria was detected in 26.6% of urine samples. Of these, the common isolates identified in catheter-associated urinary tract infection (CAUTI) and hospital-acquired urinary tract infection (HAUTI) were highly resistant to both first-line and second-line antibiotics (7). The lack of advanced diagnostic facilities and a trained workforce, a poor drug regulation system, and weak infection control mechanisms fuel urinary tract infection prevalence in developing countries (8). A similar study conducted in sub-Saharan African countries showed a 32% prevalence rate of UTI among nine countries. South Africa recorded a high UTI prevalence rate (67.7%), and the lowest UTI prevalence rate was recorded in Senegal at 5.1%. Ethiopia was found to be the fifth rank with a pooled UTI prevalence rate of 37% (9). Another study conducted on antimicrobial resistance in the East Africa region showed ampicillin, gentamicin, and ceftriaxone were highly resistant to the isolated bacteria, which puts the commonly used antibiotics at risk (10).

A study conducted in Ethiopia revealed that uropathogens with high multidrug resistance (MDR) were isolated with a prevalence rate of 88% (11). Similarly, the prevalence of multidrug-resistant bacteria in Ethiopia is high compared with much of the rest of the world, and bacterial isolates from urinary tract infections were multidrug-resistant with a prevalence rate of 67.6% (12). A study conducted among pediatric patients in Ethiopia showed that most bacterial isolates were highly resistant to commonly prescribed antibiotics. Of these, ampicillin and trimethoprim–sulfamethoxazole (TS) exhibited the highest resistance rate for tested antibiotics (13). Similarly, a study conducted in Eastern Ethiopia showed a high degree of MDR observed among the commonly used antibiotics in the area, which creates a room to revise treatment guidelines used and focus on AMR surveillance activities (14).

Urinary tract infection remains one of the most common causes of morbidity, and early diagnosis of the disease has become a crucial part of its management, as it is strongly associated with bad health outcomes and the commonly used antibiotics to treat UTI empirically are no longer functional due to sharply increasing multidrug-resistant bacterial isolates (15). In addition, the highly increasing rate of multidrug-resistant bacterial isolates in Ethiopia puts existing antibiotic treatment at risk (11). The lack of diagnostic facilities to early detect resistance types of isolates and the highly increasing resistance of bacterial isolates against antibiotics used to empirically treat UTI were growing challenges (13).

It is a known fact that disease prevalence varies in accordance with geographical settings, and it is too difficult to give the exact prevalence of both community-acquired and hospital-acquired urinary tract infections, bacterial etiologic agents, and antimicrobial resistance rates of isolates (16). It is very important to have knowledge of hospital-acquired and community-acquired bacterial isolates and antimicrobial susceptibility profiles to make first choices of antibiotics to treat the infection and, based on identified risk factors, to plan intervention measures. Even if many studies are done in the area, a comparative study on community-acquired and hospital-acquired urinary tract infections is lacking. Therefore, this study aimed to assess the comparative burden of nosocomial and community-acquired bacterial UTIs and their associated factors in patients attending Hiwot Fana Comprehensive University Hospital.

## 2 Methods and materials

### 2.1 Study area

The study was conducted at Hiwot Fana Comprehensive University Hospital, Harar, Ethiopia. Hiwot Fana Comprehensive Specialized University Hospital (HFCSUH) was established in 1941 and became a university-specialized hospital for Haramaya University's College of Health and Medical Sciences in 2010. The hospital is the largest teaching and referral hospital in Eastern Ethiopia, serving many people coming from the surrounding zones and nearby regions, both for inpatient and outpatient services. It is located 525 km from the capital city, Addis Ababa, and serves as the only cancer treatment center in Eastern Ethiopia and consists of an operating room, intensive care unit (ICU), pediatric ward, gynecology and obstetrics ward, orthopedics ward, medical ward, recovery ward, and trauma and cancer centers.

### 2.2 Study design and period

A hospital-based comparative cross-sectional study was conducted from January to April 2024.

### 2.3 Source population

All inpatient and outpatient department attendants visiting Hiwot Fana Specialized University Hospital during the study period were the source population.

### 2.4 Study population

Inpatient and outpatient attendants who were clinically suspected of urinary tract infection during the study period were the study population.

#### 2.4.1 Exclusion criteria

Symptomatic UTI patients who took antibiotics within the previous 2 weeks and who were not volunteers to participate were excluded from the study.

### 2.5 Sample size determination and sampling techniques

The sample size was determined using the Epi Info Version 7 sample size calculator with the double population proportion that was used in a previous study. The calculation resulted in a sample size of 154. Then, we assumed a 10% non-response rate. Finally, by considering the stratified analysis, we doubled the sample size to 338 (169 from each group). P1, the prevalence of community-acquired UTI, was 19.3% (17) from a previous study conducted in Dessie and P2, the prevalence of hospital-acquired UTI, was 41.5% (18) from another study conducted in Northeast Ethiopia. At a 95% confidence interval and power at 80%, n1 (number of community-acquired UTI patients) and n2 (number of hospital-acquired UTI patients) = 1:1. The final sample size was 338 (159 hospital-acquired and 159 community-acquired UTI patients). A convenient sampling technique was employed to recruit study participants consecutively until the required sample size was reached because of time constraints and the lack of a readily available sampling frame. Nosocomial UTI was determined if a patient had a clinical urinary tract infection 48 h after hospital admission, and it was not the initial reason for admission. Community-acquired UTI was assumed when a patient developed clinical urinary tract infections during his hospital visit or before 48 h of hospital admission. Clinical UTI is a condition when a patient has one or more of the following symptoms: dysuria, frequency, urgency, hematuria, back pain, nocturia, costovertebral angle tenderness, and the absence of vaginal discharge or irritation (19).

### 2.6 Data collection

Initially, patients with clinical UTIs were identified and recruited by physicians who were assigned and working in the respective inpatient and outpatient departments during the study period. A structured questionnaire-based patient interview was conducted, and the questionnaire was adopted from similar studies (15, 20) with some amendments. It was administered to collect information related to potential UTI-associated factors in the study. The questionnaires contained three parts: sociodemographic characteristics and clinical and behavioral risk factors of UTI. Two trained nurses and two laboratory technologists participated in data collection, and supervision was conducted by a health officer and senior medical microbiologist working in the hospital.

### 2.7 Laboratory investigation

#### 2.7.1 Sample collection and transportation

Study participants were oriented to cleansing the urethral opening with water and collecting freshly voided midstream urine samples of ∼10–15 ml using a sterile, wide-mouthed, screw-capped plastic container to avoid the risk of contamination. After collection, urine samples are labeled with identification number and date of collection. In catheterized patients, the soft rubber connector between the catheter and the collecting tubes is cleaned with 70% ethanol to avoid contamination. In case of delays in processing, samples were refrigerated to prevent cell decomposition and overgrowth.

#### 2.7.2 Bacterial isolation and identification method

The urine samples were taken using a standardized calibrated wire loop (0.001 ml) and inoculated onto blood agar medium (Oxoid, Hampshire, UK) and MacConkey agar medium. The streaked culture plates were incubated aerobically for 24 h at 35°C–37°C. After overnight incubation, cultured media showing significant bacterial growth were further identified by their colony morphology, gram reaction, and biochemical tests. A 0.001 ml capacity loop was used for inoculation of urine, and then the number of colony-forming units (CFU) on blood agar was multiplied by 1,000 to get organisms per milliliter. Quantification of a single bacterium >10^5^ CFU/ml growth was indicative of UTI (75).

### 2.8 Antimicrobial susceptibility testing

The antimicrobial susceptibility test was carried out by the Kirby–Bauer disc diffusion method as per the Clinical Laboratory Standards Institute (21) guidelines on Mueller–Hinton agar (Oxoid, Basingstoke, England). A bacterial suspension of each isolate was prepared in 0.5 ml of nutrient broth medium, and the turbidity was adjusted to match the 0.5 McFarland standards to obtain approximately the organism number of 1 × 10^6^ CFU/ml. A sterile cotton swab was immersed into the suspension. Then, the swab was applied to the center of the Mueller–Hinton agar plate and evenly spread on the medium (Oxoid, Basingstoke, UK). After 5 min, selected antimicrobial disks were aseptically placed on Mueller–Hinton agar plates and allowed to stand at room temperature for 15 min. The diameter of the zone of inhibition around the disk was measured using a ruler and compared to reference points stated in clinical laboratory guidelines (21), and the results were interpreted as sensitive, intermediate, and resistant.

The following antibiotics were used for this study includes as follows: gentamicin (GN) 10 µg, nitrofurantoin (F) 300 µg, tetracycline (T) 30 µg, cefazolin (Cz) 30 µg, cefoxitin (CXT) 30 µg, meropenem (MEM) 10 µg, tobramycin (TN) 10 µg, aztreonam (ATM) 30 µg, clindamycin (CN) 2 µg, rifampin (RF) 5 µg, chloramphenicol (C) 30 µg, ampicillin (AMP) 10 µg, amoxicillin–clavulanic acid (AMC) 10 µg, ceftazidime (CAZ) 30 µg, ciprofloxacin (CIP) 5 µg, trimethoprim–sulfamethoxazole (TS) 23.75 µg, erythromycin (E) 15 µg (obtained from Oxoid, Hampshire, UK) (21), and ceftriaxone (CRO).

### 2.9 Operational definitions

**Community-acquired urinary tract infection:** when patients with urinary tract infection symptoms visit the HFSUH outpatient unit or when they become symptomatic within the previous 48 h of admission (4).

**Multidrug resistance (MDR):** refers to bacteria that are resistant to at least one agent in three or more antibiotic categories (22).

**Nosocomial urinary tract infection:** when patients develop urinary tract infections in the hospital after 48 h of admission, which are not present at the time of admission (3).

**History of use of antibiotics:** UTI patients who took any antibiotic within 2 weeks before the study (23).

### 2.10 Quality control

The structured questionnaire was translated into the local languages (Afan Oromo and Amharic) and English language to check the consistency of the questions. A pilot study was conducted by pretesting translated questionnaires in the local language to make them suitable for 5% of the total sample size to ensure the validity and reliability of the data collection tools and the needed modification for the final data collection prior to the actual data collection at police hospital to assess its clarity, understandability, and simplicity. All the culture media were prepared by following the manufacturer's instructions, checked for sterility by incubating 5% of the prepared media for 24 h, observed for supporting the growth of organisms, and checked by inoculating control strains. Standard reference strains of *E. coli* (ATCC-25922), *S. aureus* (ATCC-25923), and *P. aeruginosa* (ATCC-27853) were used during culture and antimicrobial susceptibility testing.

### 2.11 Method of data analysis

Data collected were cleaned for completeness and consistency before data entry. Data were entered into EpiData 4.6 and then exported to STATA Version 16 software for analysis. During analysis, descriptive statistics, including mean, frequency, and percentage, were calculated to summarize the data as appropriate. Potential multicollinearity was also considered and tested using the variance inflation factor (VIF). Bivariable logistic regression was performed, and variables with *p* < 0.25 were eligible for the final model. The Hosmer–Lemshow goodness-of-fit test was applied for multivariable fitness. In multivariable logistic regression analysis, *p* < 0.05 adjusted odds ratio (AOR) with a 95% confidence interval (CI) was used to declare a significant association. Then, the findings of this study were interpreted and presented in the form of texts, tables, and graphs as appropriate.

### 2.12 Ethical considerations

Ethical clearance was obtained from the Institutional Health Research Review Committee (Ref. No. IHRERC/174/2023) of Haramaya University College of Health and Medical Sciences. Permission from HFCSUH was obtained through communicating with a letter of support that was written by the college. The objective, significance, benefit, risk, and procedural details of the study were explained, and informed, voluntarily, written, and signed consent was obtained from the head of the hospital and study participants. No identification or names were recorded to maintain confidentiality. Finally, data were collected after obtaining written informed consent from the participants. The culture-positive cases should be linked to the hospital for further management and care.

## 3 Results

### 3.1 Sociodemographic characteristics

A total of 332 symptomatic UTI study participants were included in this study, of which 166 were hospital-acquired and 166 were community-acquired clinical UTIs. Of these, 174 (53.6%) were females, and 158 (47.4%) were males. The age of study participants ranges from 1 to 85 years old, with a mean age of 36.33. Out of the total study participants, 103 (62%) inpatient attendants and 91 (54.8%) outpatient attendants did not attend formal education. The mean family size of study participants was 4.42, +/− 1.76498 SD (range, 1–12). Regarding the income status of study participants, the average monthly income was 3725.27 (±SD, 1755.756 ETB). The mean daily water intake of study participants was 1.59 L (SD, ±0.513) (Table 1).

**Table 1:** Sociodemographic characteristics of patients with clinical urinary tract infection in HFCSUH, Eastern Ethiopia, 2024 (*N* = 332).

Characteristics		HAUTI cases (*n* = 166)		CAUTI cases (*n* = 166)	
		Frequency	%	Frequency	%
Sex	Male	95	57.2	59	35.5
	Female	71	42.8	107	64.5
Age	0–14 years	18	10.8	6	3.6
	15–29 years	44	26.5	63	38
	30–44 years	50	30.1	43	25.9
	Greater than 45 years	54	32.5	54	32.5
Residence	Urban	44	26.5	87	52.4
	Rural	122	73.5	79	47.6
Marital status	Single	18	10.8	18	10.8
	Married	119	71.7	136	81.9
	Divorced	2		1	
	Widowed	7	4.2	5	3
	Children	20	12	6	3.6
Occupation	Farmer	55	33.1	36	21.7
	Student	29	17.5	15	9.0
	Housewife	58	34.9	84	50.6
	Others^a^	24	14.5	31	18.6
Educational status	No formal education	103	62	91	54.8
	Primary	40	2.4	30	18
	Secondary and above	23	13.8	45	27.1
Family size	1–5	123	74	127	76.5
	>5	43	26	39	23.5
Average monthly income (in ETB)	≤3,725	107	64.5	99	59.6
	>3,725	59	35.5	67	40.4
Livestock	Yes	88	53	75	45.2
	No	78	47	91	54.8

^a^ Government and private employees; NA, children <18 years of age.

### 3.2 Environmental and behavioral characteristics

Out of the 322 symptomatic UTI patients, 281 (87%) had improved water sources for domestic use, and 51 (15.8%) had unimproved water sources for domestic use. Less than half (42%) of the study participants had a daily water intake of ≤2 L/day. Regarding behavioral factors, 69 (21.4%) of study participants had a habit of delaying voiding urine voluntarily. Of the total symptomatic UTI patients, 97 (30%) had habits of shower frequency less than or equal to three times per week, and 235 (70%) of the study participants had habits of shower frequency greater than three times per week (Table 2).

**Table 2:** Environmental and behavioral factors of patients with clinical urinary tract infection in HFCSUH, Eastern Ethiopia, 2024 (*N* = 332).

Variables	Response	HAUTI cases (*n* = 166)		CAUTI cases (*n* = 166)	
		Frequency	Percent %	Frequency	Percent %
Toilet	Yes	154	92.8	153	92.2
	No	12	7.2	13	7.8
Types of latrines	Improved	57	36.8	82	53.6
	Not improved	98	63.2	71	46.4
Water source for domestic use	Improved	132	79.5	149	89.8
	Not improved	34	20.5	17	10.2
Daily water intake	≤2 L	75	45.2	61	36.7
	>2 L	91	54.8	105	63.3
Delay in voiding urine	Yes	32	19.3	37	22.3
	No	134	80.7	129	77.7
Handwashing facility	Yes	8	4.8	33	19.9
	No	158	95.2	133	80.1
Frequent genital washing after using the toilet	With water	63	90	90	83.3
	Water and soap	2	2.6	15	13.9
	Not usually wash	5	7.1	3	2.8
Shower frequency	One to three times per week	37	22.3	60	36.1
	More than three times per week	129	77.7	106	63.9
Direction of wiping	Front to back	15	22	52	49
	Either direction	56	78	54	51
Birth control	Yes	9	16.9	20	25
	No	44	83	60	75
Availability of a separate toilet in admitted wards (for inpatients only)	Yes	9	5.4		
	No	157	94.6		
Availability of handwashing facility in admission ward. (for inpatients only)	Yes	10	6		
	No	156	94		

### 3.3 Clinical characteristics

Out of the 332 patients with clinical UTIs, 35 (21.7%), 23 (13.8%), 22 (13.3%), and 65 (39.2%) had a history of admission in the last 2 months, a history of UTI in the last 6 months, a history of catheterization in the past 1 year, and chronic disease among inpatient study participants, respectively. Correspondingly, 36 (22%), 34 (21%), 10 (6%), and 35 (21.1%) had a history of admission in the last 2 months, a history of UTI in the last 6 months, a history of catheterization in the past 1 year, and chronic disease among outpatient study participants, respectively. Moreover, 45 (27.1%), 5 (3%), and 43 (25.9%) inpatient study participants had a history of catheterization, history of intubation, and surgery during the current admission, respectively. Approximately 30% (100) of the study participants had chronic diseases, of whom 49 (50%) had diabetes mellitus, followed by 15 (15%) with hypertension, 12 (11%) with cancer, and the remaining 25 (24%) with other chronic diseases. The risk factors associated with culture-confirmed bacterial UTI were history of admission in the last 2 months, history of UTI in the last 6 months, and surgery during admission, which were significantly associated with culture-confirmed bacterial UTI at *p* < 0.05 (Table 3).

**Table 3:** Clinical characteristics of symptomatic UTI patients in HFCSUH, Eastern Ethiopia, 2024 (*n* = 332).

Characteristics	Response options	HAUTI cases (*n* = 166)		CAUTI cases (*n* = 166)	
		Frequency	%	Frequency	%
Fever	Yes	87	52.4	63	38
	No	79	47.6	103	62
Vomiting	Yes	26	15.7	27	16.3
	No	140	84.3	139	83.7
Nausea	Yes	8	4.8	7	4.2
	No	158	95.2	159	95.8
Flank pain	Yes	81	48.8	130	78.3
	No	85	51.2	36	21.7
Hematuria	Yes	6	3.6	10	6
	No	160	96.4	156	94
Suprapubic pain	Yes	45	27.1	44	26.5
	No	121	72.9	122	73.5
Dysuria	Yes	66	39.8	35	21.1
	No	100	60.2	131	78.9
Urine urgency	Yes	15	9	23	13.9
	No	151	91	143	86.1
Pregnancy status for women of reproductive age group	Yes	8	14.8	14	17.2
	No	46	85.2	67	81.8
Previous admission history in the last 2 months	Yes	35	21.1	36	21.7
	No	131	78.9	130	83.3
Previous UTI history past 6 months	Yes	23	13.8	34	21
	No	143	86.2	132	79
Previous catheterization history past 1 year	Yes	22	13.3	10	6
	No	144	86.7	156	94
Chronic disease	Yes	65	39.2	35	21.1
	No	101	60.8	131	78.9
Immunecompromizing diseases	Yes	50	30.1	27	16.3
	No	116	69.9	139	83.7
Surgery during admission	Yes	43	25.9		
	No	123	74.1		
Length of admission	<3 days	88	53.1		
	3–7 days	58	34.9		
	>7 days	20	12		

### 3.4 The prevalence of bacterial urinary tract infections

**Figure 1:**
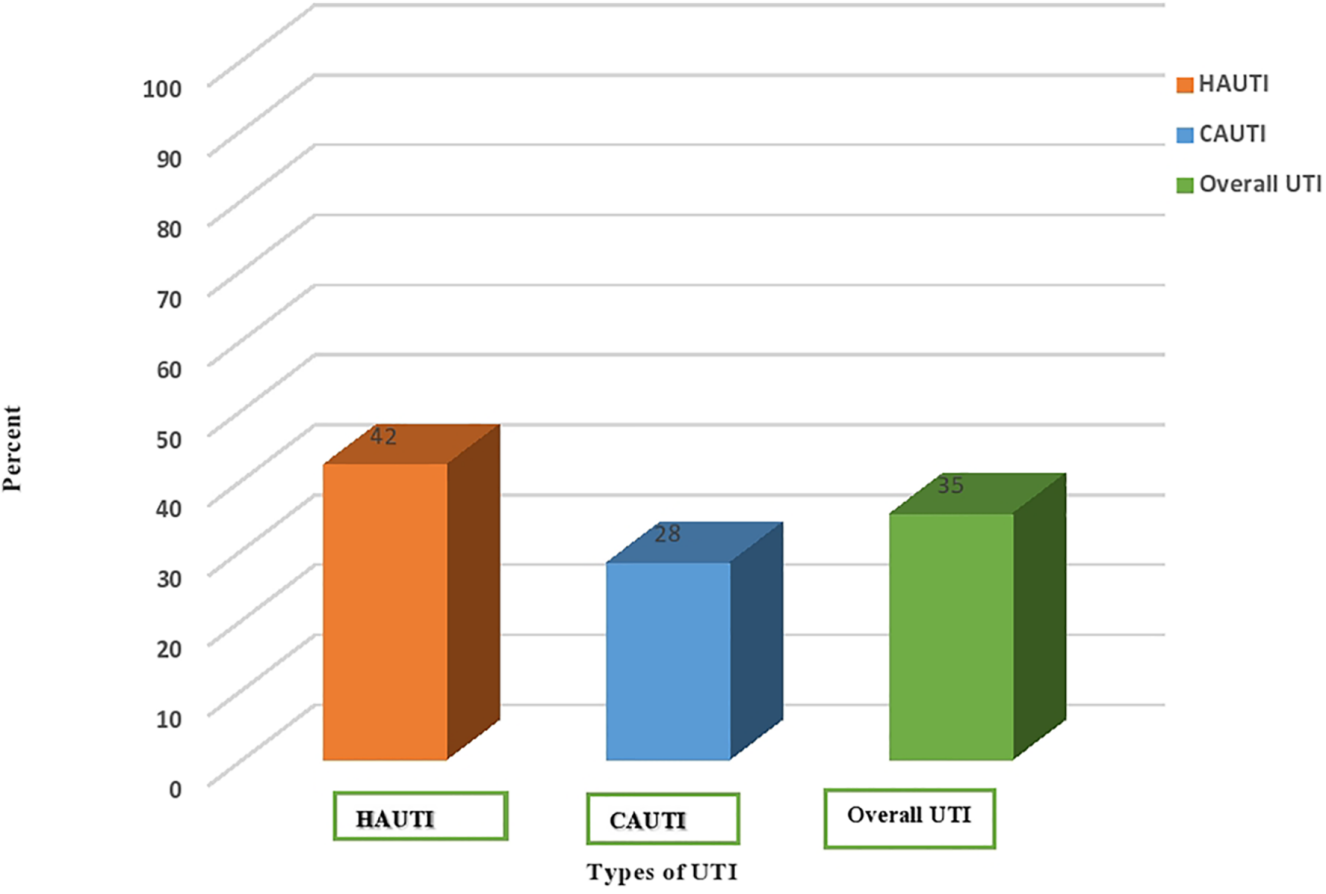
Prevalence of culture-confirmed bacterial urinary tract infection among community and nosocomial UTI cases in HFCSUH, Eastern Ethiopia, 2024. HAUTI, hospital-acquired urinary tract infections. CAUTI, community-acquired urinary tract infections.

The overall prevalence of urinary tract infection was 35% (117/332) (95% CI: 22–50). Of these, the prevalence of hospital-acquired urinary tract infection was 42% (70/166) (95% CI: 35–50), and community-acquired urinary tract infection was 28% (47/166) (95% CI: 22–36). High culture-confirmed bacterial uropathogens were isolated from rural attendants (79/201 or 39%) (Figure 1).

### 3.5 Pathogenic bacterial profiles isolated from urinary tract infections

**Figure 2:**
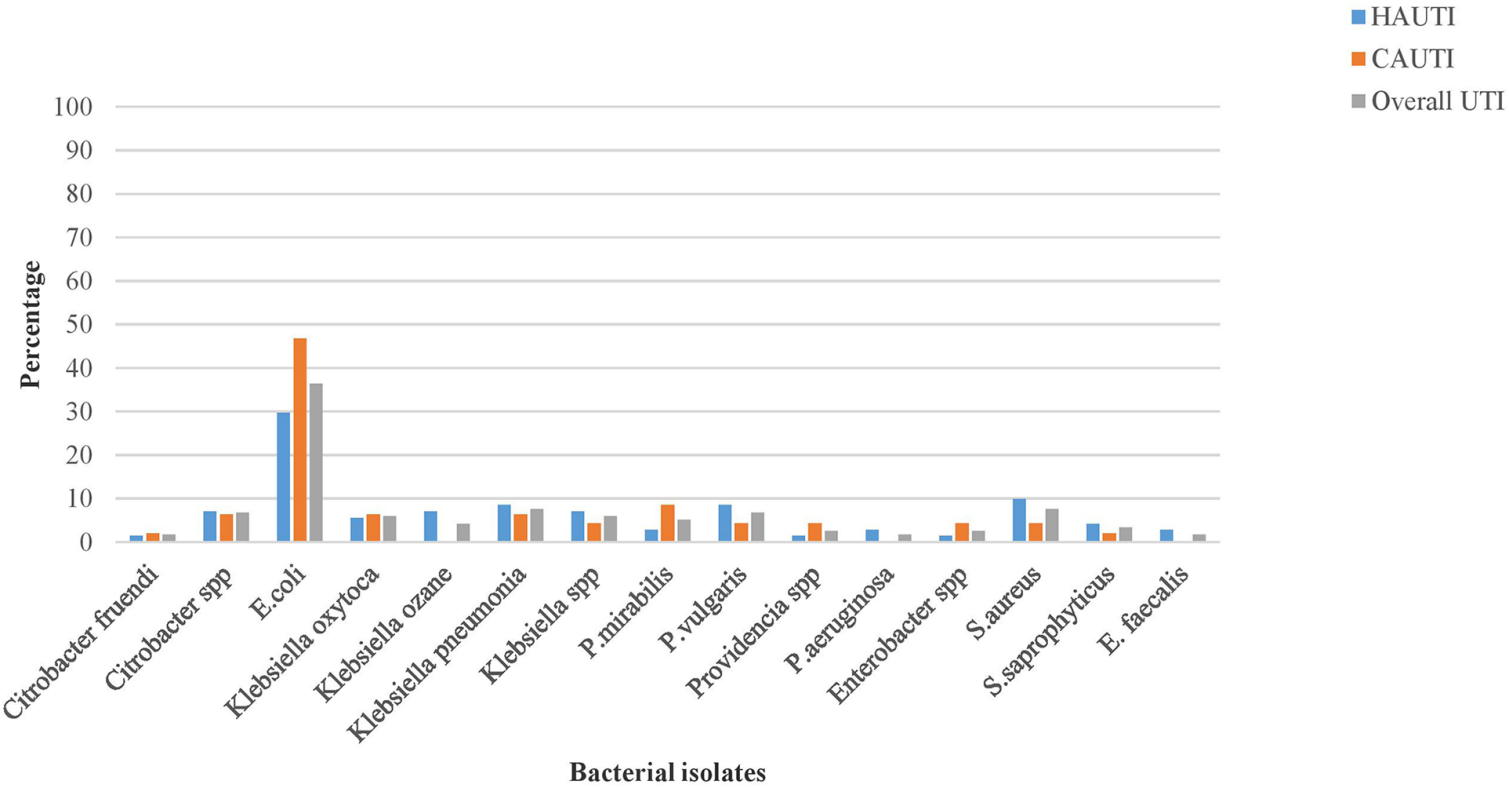
The pathogenic bacterial isolates of urine cultured among patients with clinical urinary tract infection in HFCSUH, Eastern Ethiopia, 2024.

The overall bacterial uropathogen culture-positive rate was 35%. The prevalence of hospital-acquired bacterial UTIs was 42%, whereas that of community-acquired UTIs was 28%. The majority (87%) of the isolated uropathogens responsible for UTIs were gram-negative bacteria. The predominant bacterial species isolated from hospital-acquired UTI were *E. coli* (21/71 or 29.7%) and *K. pneumoniae* (6/71 or 8.5%), whereas *E. coli* (22/47 or 46.8%) and *P. mirabilis* (4/47 or 8.5%) were the predominant bacterial species isolated from community-acquired UTIs. Among gram-positive bacteria, the predominant isolate in hospital and community-acquired UTIs were *Staphylococcus aureus*, followed by *Staphylococcus saprophyticus*, with an isolation rate of 9/118 (7.6%) and 4/118 (3.4%), respectively. It was to be noted that mixed bacterial growth was observed in one patient with an isolation rate of 1/118 (0.85%) (Figure 2).

### 3.6 Antimicrobial susceptibility patterns of bacterial isolates

Antimicrobial susceptibility tests were performed for each bacterial isolate on Muller–Hinton agar, and the susceptibility results were interpreted based on CLSI guidelines 2022. Gram-negative bacterial species isolated from hospital-acquired UTI patients showed susceptibility rates of 52 (88%), 36 (61%), and 32 (54%) to meropenem, ciprofloxacin, and gentamicin and gram-negative bacterial species isolated from community-acquired UTI patients showed susceptibility rates of 43 (98%), 29 (66%), and 24 (58%) to meropenem, ciprofloxacin, and augmentin, respectively.

Among the predominant bacterial uropathogens isolated from hospital and community-acquired UTI patients, *E*. *coli* showed higher resistance rates of 90.7% and 88.4% to tetracycline and ampicillin, respectively*.* In contrast, *E. coli* demonstrated the highest susceptibility rates to meropenem and ciprofloxacin with 88.4% and 67%, respectively. *K. pneumoniae* is the second most predominant bacterial isolate and showed resistance rates of 88.9% and 77.7% to ceftriaxone and cotrimoxazole, respectively. It also presented higher susceptibility rates or 77.8% to meropenem and 66.7% to augumentin. Gram-positive bacteria species showed the highest susceptibility rates of 86.7% and 66.7% to nitrofurantoin and ciprofloxacin, respectively, whereas gram-positive bacterial species showed the highest resistance rates of 80% and 73% to penicillin and tetracycline, respectively. At the species level, *Staphylococcus aureus* demonstrated the highest resistance rates of 100% and 88.8% to penicillin and tetracycline and the highest susceptibility rates of 77.8% and 55.6% to nitrofurantoin and gentamicin, respectively (Tables 4, 5).

**Table 4:** Antimicrobial susceptibility patterns of bacterial isolates from urine cultures among patients with nosocomial UTIs in HFCSUH, Eastern Ethiopia, 2024.

Bacterial isolates	ASP	Antimicrobial agents											
		AMP	AUG	CRO	CIP	NA	SXT	GN	MEM	F	T	CXT	P
Gram-negative bacterial isolates													
*C. fruendi*, 1 (1.4%)	S	ND	ND	1 (100)	1 (100)	1 (100)	0 (0)	0 (0)	1 (100)	0 (0)	ND		
	R			0 (0)	0 (0)	0 (0)	1 (100)	1 (100)	0 (0)	1 (100)			
Other *Citrobacter* spp., 5 (7%)	S	0 (0)	2 (0)	3 (60)	3 (60)	3 (60)	1 (20)	3 (60)	4 (80)	4 (80)	0 (0)		
	R	5 (100)	3 (0)	2 (40)	2 (40)	2 (40)	4 (80)	2 (40)	1 (20)	1 (20)	5 (100)		
*E. coli*, 21 (29.8%)	S	2 (10)	10 (48)	5 (24)	14 (67)	10 (48)	2 (10)	11 (52)	17 (81)	10 (48)	2 (10)		
	R	19 (90)	11 (52)	16 (76)	7 (33)	11 (52)	19 (90)	10 (48)	4 (19)	11 (52)	19 (90)		
*Enterobacter* spp., 1 (1.4%)	S	0 (0)	1 (100)	1 (100)	1 (100)	1 (100)	1 (100)	1 (100)	1 (100)	0 (0)	0 (0)		
	R	1 (100)	0 (0)	0 (0)	0 (0)	0 (0)	0 (0)	0 (0)	0 (0)	1 (100)	1 (100)		
*K. oxytoca*, 4 (5.6%)	S	ND	0 (0)	1 (25)	4 (100)	2 (50)	1 (25)	2 (50)	4 (100)	2 (50)	ND		
	R		4 (100)	3 (75)	0 (0)	2 (50)	3 (75)	2 (50)	0 (0)	2 (50)			
*K. ozane*, 5 (6.7%)	S	ND	2 (40)	2 (40)	3 (60)	3 (60)	2 (40)	3 (60)	5 (100)	3 (60)	ND		
	R		3 (60)	3 (60)	2 (40)	2 (40)	3 (60)	2 (40)	0 (0)	2 (40)			
*K. pneumoniae*, 6 (8.6%)	S	ND	3 (50)	0 (0)	3 (50)	1 (17)	2 (33)	2 (33)	4 (67)	3 (50)	ND		
	R		3 (50)	6 (100)	3 (50)	5 (83)	4 (67)	4 (67)	2 (33)	3 (50)			
Other *Klebsella* spp., 5 (7%)	S	ND	1 (20)	2 (40)	2 (40)	2 (40)	1 (20)	4 (80)	5 (100)	3 (60)	ND		
	R		4 (80)	3 (60)	3 (60)	3 (60)	4 (80)	1 (20)	0 (0)	2 (40)			
*P. mirabilis*, 2 (2.8)	S	0 (0)	1 (50)	1 (50)	1 (50)	1 (50)	0 (0)	1 (50)	2 (100)	ND	0 (0)		
	R	2 (100)	1 (50)	1 (50)	1 (50)	1 (50)	2 (100)	1 (50)	0 (0)		2 (100)		
*P. vulgaris*, 6 (8.5%)	S	ND	3 (50)	2 (33)	4 (67)	2 (33)	1 (17)	3 (50)	6 (100)	ND	ND		
	R		3 (50)	4 (67)	2 (33)	4 (67)	5 (83)	3 (50)	0 (0)				
*Providencia* spp. , 1 (1.4%)	S	ND	ND	1 (100)	0 (0)	0 (0)	0 (0)	1 (100)	1 (100)	1 (100)	ND		
	R			0 (0)	1 (100)	1 (100)	1 (100)	0 (0)	0 (0)	0 (0)			
*P. aeruginosa*, 2 (2.8%)	S	ND	ND		0 (0)			1 (50)	2 (100)		ND		
	R				2 (100)			1 (50)	0 (0)				
Total, 59 (83%)	S	2 (7)	22 (40)	19 (33)	36 (61)	26 (46)	11 (19)	32 (54)	52 (88)	26 (53)	2 (7)		
	R	27 (93)	33 (60)	38 (67)	23 (39)	31 (54)	46 (81)	27 (46)	7 (12)	23 (47)	27 (93)		
Gram-positive bacterial isolates													
*S. aureus*, *7* (10%)	S				3 (43)		2 (29)	4 (57)		5 (71)	1 (14)	2 (29)	0 (0)
	R				4 (57)		5 (71)	3 (43)		2 (29)	6)86)	5 (71)	7 (100)
*S. saprophyticus*, 3 (4.2)	S				2 (67)		0 (0)	2 (67)		3 (100)	1 (33)	3 (100)	1 (33)
	R				1 (33)		3 (100)	1 (33)		0 (0)	2 (67)	0 (0)	2 (67)
*E. faecalis*, 2 (2.8%)	S				2 (100)		2 (100)	1 (50)		2 (100)	1 (50)	2 (100)	1 (50)
	R				0 (0)		0 (0)	1 (50)		0 (0)	1 (50)	0 (0)	1 (50)
Total, 12 (17%)	S				7 (58)		4 (33)	7 (58)		10 (83)	3 (25)	7 (58)	2 (17)
	R				5 (42)		8 (67)	5 (42)		2 (17)	9 (75)	5 (42)	10 (83)

ASP, antimicrobial susceptibility pattern; S, susceptible; R, resistant; AMP, ampicillin; AUG, augmentin; CRO, ceftriaxone; CIP, ciprofloxacin, NA, nalidixic acid; STX, cotrimoxazole; GN, gentamicin; MEM, meropenem; F, nitrofurantoin; T, tetracycline; CXT, cefoxitin; P, penicillin.

**Table 5:** Antimicrobial susceptibility patterns of bacterial isolates from urine cultures among patients with community-acquired UTIs in HFCSUH, Eastern Ethiopia, 2024.

Bacterial isolates	ASP	Antimicrobial agents											
Gram-negative bacterial isolates													
		AMP	AUG	CRO	CIP	NA	SXT	CN	MEM	F	T	CXT	*P*
*C. fruendi*, 1 (2%)	S	ND	ND	0 (0)	0 (0)	1 (100)	0 (0)	0 (0)	1 (100)	0 (0)	ND		
	R			1 (100)	1 (100)	0 (0)	1 (100)	1 (100)	0 (0)	1 (100)			
Other *Citrobacter* spp., 3 (6.4%)	S	0 (0)	1 (33)	0 (0)	1 (33)	1 (33)	1 (33)	0 (0)	3 (100)	2 (67)	0 (0)		
	R	3 (100)	2 (66)	3 (100)	2 (67)	2 (67)	2 (67)	3 (100)	0 (0)	1 (33)	3 (100)		
*E. coli*, 22 (46.8%)	S	2 (10)	16 (73)	5 (23)	15 (68)	13 (59)	4 (18)	9 (41)	21 (95)	10 (41)	3 (14)		
	R	19 (90)	6 (27)	17 (77)	7 (32)	9 (41)	18 (82)	13 (59)	1 (5)	12 (59)	19 (86)		
*Enterobacter* spp., 2 (4.3%)	S	0 (0)	1 (50)	0 (0)	2 (100)	1 (50)	0 (0)	2 (100)	2 (100)	1 (50)	0 (0)		
	R	2 (100)	1 (50)	2 (100)	0 (0)	1 (50)	2 (100)	0 (0)	0 (0)	1 (50)	2 (100)		
*K. oxytoca*, 3 (6.4%)	S	ND	1 (33)	2 (67)	3 (100)	2 (67)	0 (0)	1 (33)	3 (100)	1 (33)	2 (67)		
	R		2 (67)	1 (33)	0 (0)	1 (33)	3 (100)	2 (67)	0 (0)	2 (67)	1 (33)		
*K. pneumonia*, *3* (6.4%)	S	ND	3 (100)	1 (33)	2 (67)	2 (67)	0 (0)	1 (33)	3 (100)	3 (100)	ND		
	R		0 (0)	2 (67)	1 (33)	1 (33)	3 (100)	2 (67)	0 (0)	0 (0)			
Other *Klebsella* spp., 2 (4.3%)	S	ND	0 (0)	1 (50)	2 (100)	0 (0)	0 (0)	1 (50)	2 (100)	2 (100)	1 (50)		
	R		2 (100)	1 (50)	0 (0)	2 (100)	2 (100)	1 (50)	0 (0)	0 (0)	1 (50)		
*P. mirabilis*, 4 (8.5%)	S	0 (0)	1 (25)	0 (0)	3 (75)	3 (75)	1 (25)	3 (75)	4 (100)	ND	ND		
	R	4 (100)	3 (75)	4 (100)	1 (25)	1 (25)	3 (75)	1 (25)	0 (0)				
*P. vulgaris*, 2 (4.3%)	S	ND	2 (100)	0 (0)	1 (50)	1 (50)	1 (50)	0 (0)	2 (100)	ND	ND		
	R		0 (0)	2 (100)	1 (50)	1 (50)	1 (50)	2 (100)	0 (0)				
*Providencia* spp., 2 (4.3%)	S	ND	ND	0 (0)	2 (100)	2 (100)	1 (50)	1 (50)	2 (100)	2 (100)	1 (50)		
	R			2 (100)	0 (0)	0 (0)	1 (50)	1 (50)	0 (0)	0 (0)	1 (50)		
Total, 44 (93.7%)	S	3 (10)	24 (58)	8 (19)	29 (66)	21 (48)	7 (16)	15 (34)	43 (98)	20 (53)	7 (19)		
	R	28 (90)	17 (42)	36 (91)	15 (34)	23 (52)	37 (84)	29 (66)	1 (2)	18 (47)	30 (81)		
Gram-positive isolates													
*S. aureus*, 2 (4.3%)	S				2 (100)		1 (50)	1 (50)		2 (100)	0 (0)	2 (100)	0 (0)
	R				0 (0)		1 (50)	1 (50)		0 (0)	2 (100)	0 (0)	2 (100)
*S. sapropyticus*, 1 (2%)	S				1 (100)		0 (0)	1 (100)		1 (100)	1 (100)	0 (0)	1 (100)
	R				0 (0)		1 (100)	0 (0)		0 (0)	0 (0)	1 (100)	0 (0)
Total, 3 (6.3%)	S				3 (100)		1 (33)	2 (67)		3 (100)	1 (33)	2 (67)	1 (33)
	R				0 (0)		2 (67)	1 (33)		0 (0)	2 (67)	1 (33)	2 (67)

ASP, antimicrobial susceptibility pattern; S, susceptible; R, resistant; AMP, ampicillin; AUG, augmentin; CRO, ceftriaxone; CIP, ciprofloxacin; NA, nalidixic acid; STX, cotrimoxazole; GN, gentamicin; MEM, meropenem; F, nitrofurantoin; T, tetracycline; CXT, cefoxitin; P, penicillin.

### 3.7 Multidrug resistance patterns of bacterial isolates

The overall MDR rate of the bacterial isolate was 77.8%, and a higher MDR rate was observed among gram-negative bacterial isolates, with a rate of 78.6%. *E. coli*, *K. pneumoniae*, and *Citrobacter* spp. isolates showed MDR rates of 39 (37.8%), 8 (7.8%), and 7 (6.8%), respectively, whereas *Pseudomonas aeruginosa* demonstrated the lowest MDR rate of 0%. Among gram-positive bacterial isolates, *Staphylococcus aureus* revealed a higher MDR rate of 7 (40%) for tested antibiotics in the panel, and *Enterococcus faecalis* isolates had the lowest MDR rate of 1 (6.7%) (Table 6).

**Table 6:** Multidrug resistance patterns of bacterial isolates from urine cultures of patients with clinical UTIs in HFCSUH, Eastern Ethiopia, 2024.

Bacterial isolates *n* (%)	Resistance pattern						
	RO	R1	R2	R3	R4	≥ R5	MDR
*Ctrobacter fruendi*, 2 (1.7%)	0	0	1	0	1	0	1
*Citrobacter* spp., 8 (6.8%)	0	0	1	1	2	4	7
*E. coli*, 43 (36%)	0	0	4	7	6	26	39
*Entrobacter* spp. , 3 (2.5%)	0	1	0	1	1	0	2
*K. oxytoca*, 7 (5.9%)	0	0	1	0	3	3	6
*Klebsiella ozane*, 5 (4.8%)	0	0	2	1	0	2	3
*Klebsiella pneumonia*, 9 (8.7%)	0	0	1	0	0	8	8
*Klebsiella* spp., 7 (5.9%)	0	1	1	0	0	5	5
*Proteus mirabilis*, 6 (5.1%)	0	0	0	3	1	2	5
*Proteus vulgaris*, 8 (6.8%)	0	0	4	1	0	3	4
*Providencia* spp., 3 (2.5%)	0	0	2	0	1	0	1
*Pseudomonas aeruginosa*, 2 (1.7%)	0	1	1	0	0	0	0
*Staphylococcus aureus*, 9 (7.6%)	0	0	1	1	1	4	6
*Staphylococcus saprophyticus*, 4 (3.4%)	0	0	2	1	0	1	2
*Entercoccus faecalis*, 2 (1.7%)	0	0	2	1	0	0	1
Total, 118 (100)	0	3	23	17	16	58	91 (77%)

R0 = no antibiotic resistance; R1 = resistance to one antibiotic; R2 = resistance to two antibiotics; R3 = resistance to three antibiotics; R4 = resistance to four antibiotics; ≥ R5 = resistance to five or above antibiotics.

### 3.8 Factors associated with urinary tract infections

In bivariate analysis, those variables with *p* < 0.25 were selected as candidates for the multivariable analysis. In multivariable logistic regression analysis, surgery during admission, delay in voiding urine, previous UTI history, and previous admission history were significantly associated with culture-confirmed bacterial UTI at *p* < 0.05.

Patients who had not undergone surgery during admission had a 98% (AOR, 0.02; 95% CI: 0.00002–0.3) lower risk of developing culture-confirmed bacterial UTI compared to their counterparts. Patients who had no previous history of UTI had a 96% (AOR, 0.04; 95% CI: 0.004–0.4) lower risk of developing culture-confirmed bacterial UTI compared to those with a previous history of UTI in the last 6 months, and patients who had no previous admission history in the last 2 months had a 93% (AOR, 0.07; CI: 0.01–0.5) lower risk of developing culture-confirmed bacterial UTI. Patients with educational status of secondary and above had a 98% (AOR, 0.02; 95% CI: 0.001– 0.6) lower risk of developing bacterial UTI compared to patients who had primary or no formal education (Table 7).

**Table 7:** Bivariable and multivariable logistic regression analysis of risk factors of bacterial UTI among patients with clinical UTIs in HFCSUH, Eastern Ethiopia, from January 19 to April 19, 2024.

Variables		Culture-positive bacterial HAUTIs			*p*-value	Culture-positive bacterial CAUTIs			*p*-value
		Negative	Positive	AOR (95% CI)		Negative	Positive	AOR (95% CI)	
Education	No formal education	51	52	1		55	36	1	
	Primary	29	11	0.08 (0.002–3.2)	0.181	24	6	0.1 (0.005–2.2)	0.144
	Secondary & above	16	7	14.6 (0.1–19.1)	0.173	40	5	0.02 (0.001–0.6)	0.024*
Family size	1–5	77	46	1		94	33	1	
	>5	19	24	2.4 (0.1–85.6)	0.628	25	14	0.8 (0.1–9.4)	0.866
Monthly income	≤3,725 ETB	55	52			65	34		
	>3,725 ETB	41	18	1.9 (0.1–35.7)	0.675	54	13	0.1 (0.01–1.2)	0.075
Length admission	<72 h	17	1						
	72 h–1 week	74	54						
	>1week	5	15						
Catheterization during admission	Yes	13	32						
	No	83	38	0.2 (0.01–3.9)	0.300				
Surgery during admission	Yes	16	27						
	No	80	43	0.02 (0.00002–0.3)	0.016*				
Previous admission history in the last 2 months	Yes	8	27			17	19		
	No	88	43	0.06 (0.002–1.7)	0.101	102	28	0.07 (0.01–0.5)	0.007*
Previous UTI history in the last 6 months	Yes	5	20			15	19		
	No	91	50	0.7 (0.001–4.0)	0.196	104	28	0.04 (0.004–0.4)	0.005*
Chronic disease	Yes	29	36			17	18		
	No	67	34	0.7 (0.01–54.1)	0.885	102	29	0.05 (0.00002–108.4)	0.445
Immunocompromising disease	Yes	22	28			13	14		
	No	74	42	1.4 (0.02–89.8)	0.864	106	33	0.7 (6.3–7.5)	0.940
Livestock	Yes	58	30			54	21		
	No	38	40	10.2 (0.5–192.0)	0.120	65	26		
Direction of wiping	Front to back	10	5			41	11		
	Either direction	21	35	35.8 (0.2–0.7861)	0.193	31	24	0.4 (0.04–3.7)	0.990
Delay in voiding urine	Yes	7	25			14	23		
	No	89	45			105	24	0.01 (0.0005–0.1)	0.001*
Shower frequency	One to three times per week	30	7			52	8		
	>3 times per week	66	63	29.5 (1.1–787.8)	0.042*	67	39	0.05 (0.1–3.9)	0.483
Water source for domestic uses	Improved	86	46			114	35		
	Unimproved	10	24	0.3 (0.008–9.7)	0.486	5	12	0.9 (0.13–0.355	0.486
Underwear changing	Once a week	67	37			93	30		
	At least once a week	29	33	0.4 (0.02–7.4)	0.533	26	17	0.006 (0.00014–0.3)	0.009*
Daily water intake	≤2l	39	36			31	30		
	>2l	57	34	16.5 (1.9–284.3)	0.053	88	17	0.006 (0.0003–0.1)	0.001*

AOR, adjusted odds ratio; COR, crude odds ratio.

* Statistically significant at *p* < 0.25 in the bivariate analysis.

** Statistically significant at *p* < 0.05 in the multivariable analysis.

## 4 Discussions

This study showed an overall UTI prevalence rate of 35% (95% CI: 22–50), which was in line with a study conducted in local areas showing prevalence rates of 31% (20) and 31.8% (24). This finding was lower than those of the previous local studies, with UTI prevalence rates of 90% (25) and 65% (26). Although it was not statistically significant, the current study found a higher prevalence rate of hospital-acquired UTIs, 42% (95% CI: 35–50), compared to community-acquired bacterial UTIs, 28% (95% CI: 22–36). These findings are concordant with the results of other similar studies (27, 28). This could be due to several factors, including the fact that hospitalized patients are usually catheterized, have delayed urine voiding, lack urine meter drainage, and have underlying diseases such as diabetes mellitus. Additionally, factors such as microbial contamination of the drainage bag, intrahospital transfers, cross-infections in overcrowded patient rooms, immunosuppressive therapy, and surgical operations contribute to the risk (29–31).

The current prevalence of nosocomial UTI was concurrent with the previous studies conducted in Northeast Ethiopia (41.5%) (18) and Somalia (42.9%) (32). However, this finding is higher than those of the studies conducted in Jimma, Ethiopia (25.4%) (33), and Egypt (18.9%) (34). This high prevalence of hospital-acquired urinary tract infection might be due to deprived infection control measures implemented in the hospital settings of the study (35). Our study also pointed out a community-acquired UTI prevalence rate of 28% (95% CI: 22–36). This finding is comparable with those of the studies conducted in Dar es Salaam, Tanzania (27.4%) (36), and Iraq (27.3%) (37). Our finding is higher than those of most previous studies conducted in Northwest Ethiopia (19.3%) (27) and Dhaka (20%) (38). This high prevalence of community-acquired urinary tract infections might be due to poor environmental and personal hygiene, variation in the study population, and geographical locations (39).

The study also revealed the predominant bacterial uropathogen responsible for urinary tract infections was gram-negative bacteria, accounting for a rate of 103 (87%). Our study was consistent with studies conducted in other parts of the world that showed the dominant causative agents were gram-negative bacteria (15, 38, 40, 41). Of the isolates, the commonest bacterial uropathogen recovered in both hospital and community-acquired UTIs was *E*. *coli*, with rates of 21 (30.4%) and 22 (46.8%), respectively. This finding was in agreement with the findings of studies conducted in different countries revealing that *E. coli* remains the leading causative agent of bacterial uropathogens in both community and hospital-acquired UTIs (27, 42, 43).

The predominance of *Escherichia coli* as causative agents of UTI might be due to its structural flagella and pili, which enabled it to move, and dominating normal flora in the epithelium of the intestine resulted in major UTI occurrence (44). The study also revealed that *Klebsiella pneumonia* was the second most common (6; 8.7%) bacterial pathogen in hospital-acquired UTIs while *Proteus mirabilis* (4; 8.5%) was common in community-acquired UTIs. This was in agreement with studies conducted in Addis Ababa (11; 7.8%) (20) and Tigray (22.4%) (45). This study contradicted some of the local studies where *Staphylococcus aureus* was the second most predominant bacterial isolate recovered in hospital and community-acquired UTIs (27). These could be due to the fact that most gram-negative bacteria (GNB) are gut residents that colonize the urethra and subsequently the bladder through the action of specific adhesins and then begin to multiply, producing toxins and enzymes that promote their survival and could have significant clinical importance that lead to high morbidity and mortality (46).

The present study revealed that gram-negative bacterial species isolated from both groups showed a high level of resistance to ampicillin and tetracycline with rates of 91.6% and 86% and a high level of susceptibility to meropenem and ciprofloxacin with rates of 92.2% and 63%. This high resistance rate of gram-negative bacterial isolates to ampicillin was comparable with other similar studies (15, 47). These highest resistance rates of gram-negative bacteria isolates to ampicillin and tetracycline might be due to the easy accessibility of the drugs at the community and facility levels, which are prone to misuse by patients and health workers. On the other hand, the gram-negative uropathogens isolated in our study were more susceptible (92%) to meropenem, and it was congruent to the previous studies conducted in Addis Ababa, 91% (13), and in Jimma, 96% (33). *E. coli* showed the highest levels of susceptibility to meropenem with a rate of 88% and ciprofloxacin with a rate of 67%. In contrast, *E. coli* showed a higher resistance level (88%) to ampicillin and tetracycline. This high resistance rate of *E. coli* to tetracycline was comparable with a study (45), and the extrusion of drugs from the cytoplasm via specific efflux helped pump tetracycline (48).

The present study showed that the overall MDR prevalence rate is 77.8%, which is higher than those of the studies in Gondar (70.4%) (49), Eastern Ethiopia (61.6%) (47), Iran (56%) (41), and Bangladesh (72%) (50), and Northwest Ethiopia (55.7%) (24). These variations could be due to the irrational use of drugs at community levels (36). The current high MDR rate could resulted from the muddled use of antimicrobial agents in the study area (51), and it emphasizes the need for a strong antimicrobial stewardship implementation program that will lead to effective AMR prevention and control. This study also indicated nearly 3/4 of the MDR prevalence rate among gram-negative bacteria isolates, and this finding was in agreement with previous studies done elsewhere (38, 52). The reason for the high MDR prevalence among gram-negative bacteria might be the majority of the groups had intrinsic resistance and most of the bacterial isolates were acquired in hospital settings (53). Of the gram-positive bacterial isolates, *Staphylococcus aureus* showed the highest MDR rate of 40%, whereas *E. faecalis* from gram-positive and *P. aeruginosa* from gram-negative bacterial isolates showed the lowest MDR rate. These findings were discordant with similar studies due to variations in study areas and community.

In the current study of outpatients who had secondary or above educational status by far, 98% had a lower risk of UTIs compared to those who never went to formal education. The finding is in line with other similar studies in Ethiopia (14), confirming that education increases knowledge and improves most modifiable individual-level factors with a reduction in the urinary tract infection rate (54). Similar results were observed in other studies (55–57). This study showed that patients who had previous UTI history within the last 6 months were 96% at higher risk of developing UTI compared to those who had no UTI history in the timeline, which was supported by other studies (20, 24, 58, 59). A history of previous UTIs increases the risk of developing a new active UTI because it can alter the bladder lining, making it more susceptible to bacterial colonization, potentially due to changes in the immune response triggered by the initial infection. This allows bacteria to more easily adhere and establish themselves, leading to recurrent infections (60). In our study, patients who had no previous admission history within the previous 2 months had a 93% lower risk of developing bacterial UTI compared to their counterparts. The study corresponds to studies conducted in southern Ethiopia (15), Pakistan (26), and Uganda (4). The epidemiology and presentation of UTIs are changing over time in patients with frequent hospitalization (61).

The study also showed that outpatients who were delayed in voiding urine habits had a 99% higher risk of contracting bacterial UTIs than their counterparts. This finding is in agreement with similar studies conducted in different areas (15, 62), and delaying or holding urine for a long time increases the chances of UTIs because it allows bacteria to multiply in the bladder due to urine stasis, which is when urine remains in the bladder for too long; essentially, the longer you hold your urine, the higher the chance of developing a UTI (63). Moreover, this study also indicated that outpatients who changed their underwear at least once a week were 94% less likely to have a UTI than those who did not change their underwear within a week. This result was in agreement with a similar study (64). Infrequent underwear changing often deprives personal hygiene and favors the ascending of bacterial uropathogens into upper urinary tracts from the genital area, resulting in microbial colonization and multiplication, ending up in a complicated urinary tract infection (65).

The current study also showed that patients whose daily water intake was >2 L per day were 96% less likely to develop UTI compared to those who took ≤2 L of daily water. This is a fundamental established truth supported by various studies (66, 67), and high daily water intake reduces invasion of the urinary tract by flushing the urinary tract and makes an unfavorable environment for multiplication (68).

Inpatients who had not undergone surgery during admission had a 98% lower likely risk of developing bacterial UTIs compared to those who had surgery during admission. This finding is consistent with other studies conducted in different areas (69–71). Routine catheterization has been employed in many surgical centers to avoid postoperative urinary retention, something for which patients undergoing total joint arthroplasty are known to be at increased risk and which itself is associated with UTIs (72). This study also indicated that inpatients who took showers greater than three times per week had ∼30 times lower risk of getting a UTI compared to those who took showers three or fewer times per week. This finding was also comparable with another study (73). That is why lack of access to the necessary hygiene facilities resulted in inadvertent health outcomes and UTIs (74). In our study, some sociodemographic and behavioral risk factors that commonly had significant associations with bacterial UTIs in many other studies did not demonstrate statistically significant associations with either the hospital- or community-acquired UTIs. The potential reasons could be the differences in study population type, socioeconomic status, and geography.

### 4.1 Limitations

The study employed a non-probability sampling method.

## 5 Conclusions

The present study revealed that the rate of hospital-acquired UTIs is relatively higher than that of community-acquired ones. *E. coli*, *S. aureus*, *K. pneumoniae*, and *P. mirabilis* were the prominent uropathogens isolated from hospital-acquired and community-acquired urinary tract infections. *E. coli* showed the highest resistance rates to tetracycline and ampicillin and the highest susceptibility rates to meropenem and ciprofloxacin. The study also revealed that there was a very high MDR rate in bacterial UTIs in the study area. Generally, modifiable individual-level factors such as educational status, delay in voiding urine habits, previous UTI history within the last 6 months, admission history in the previous 2 months, and infrequent changing of underwear were the significantly associated factors that play a role in community-acquired UTIs, whereas surgery during admission and taking infrequent showers at the time of admission were the main risk factors for hospital-acquired UTIs. Therefore, increasing community awareness and knowledge about UTIs and their treatment through health education is mandatory to reduce both types of UTIs. Strengthening the infection prevention and control implementation strategies regularly could have an immense and indispensable impact in reducing hospital-acquired UTIs. Clinicians and other stakeholders should advocate for a conservative and proper use of antibiotics. The MDR challenge demands regular surveillance, and the application of updated antibiograms is crucial to monitor the AMR situation in the country. Finally, the government and non-government organizations should collaborate to put in place an integrated AMR surveillance system at all levels of hospitals in Ethiopia.

## Data Availability

The original contributions presented in the study are included in the article/Supplementary Material; further inquiries can be directed to the corresponding author.
